# An Experimental and Computational Study on the Effects of Ball Milling on the Physicochemical Properties and Digestibility of a Canna Starch/Tea Polyphenol Complex

**DOI:** 10.3390/foods14020208

**Published:** 2025-01-10

**Authors:** Yizhou Wang, Kejun Di, Ying Sun, Xiaojing Li, Jiong Zheng, Fusheng Zhang

**Affiliations:** 1 College of Food Science, Southwest University, Chongqing 400715, China; 17723945045@163.com (Y.W.); dikejun4@163.com (K.D.); sunying991215@163.com (Y.S.); lxj001029@163.com (X.L.); zhengjiong_swu@126.com (J.Z.); 2 Chongqing Key Laboratory of Speciality Food Co-Built by Sichuan and Chongqing, Chongqing 400715, China

**Keywords:** ball milling, tea polyphenols, interactions, digestibility, molecular dynamics simulations

## Abstract

To investigate the impact of tea polyphenols on the thermodynamic properties, gelatinization properties, rheological properties, and digestion characteristics of starch after ball milling, canna starch and tea polyphenols were mixed at a 10:1 ratio (*w*/*w*) in an experiment and processed with different ball milling times. After ball milling for 3 h, the tea polyphenols and starch fragments formed complexes. Compared with the unmilled mixture, the solubility increased by 199.4%; the shear stress decreased by 89.48%; and the storage modulus and loss modulus decreased. The content of resistant starch first decreased and then increased. Infrared results revealed that ball milling led to a non-covalent interaction between the tea polyphenols and starch. Molecular dynamics simulations were used to study the interaction between the starch and tea polyphenols. The binding free energy of the main component, epigallocatechin gallate (EGCG), in tea polyphenols with starch was reduced from −23.20 kcal/mol to −26.73 kcal/mol. This experiment can provide a reference for the development of functional starch with high resistant starch content.

## 1. Introduction

Starch, an important daily source of carbohydrates, is closely related to an increase in blood sugar levels after meals [1]. As an important source of nutrients, starchy foods are hydrolyzed by enzymes and converted into small glucose molecules to enter the bloodstream and supply energy for the human body [2], but a rapid increase in the blood glucose concentration greatly increases the risk of obesity and diabetes. The development of starch products with low digestibility has the potential to thereby significantly attenuate the postprandial glycemic response and promote the formation of resistant starch (RS) [3]. RS can undergo fermentation by the gut microbiome, leading to the production of health-promoting metabolites such as short-chain fatty acids (SCFAs), which contribute to gastrointestinal well-being [4,5]. In addition, the digestibility of starch can be diminished by modifying the structure of starch or other food constituents, impeding the interaction between starch and enzymes or inhibiting amylase activity. Starch with higher content of short and medium amylose chains exhibits lower digestibility after cooking [6]. Therefore, this study focused on ensuring the nutritional properties of starch and increasing the content of RS by altering the structure of starch and adding other food components, thereby enhancing the resistance to digestion.

Tea polyphenols (TPs) are a group of polyphenolic compounds present in tea. They slow down the rate of enzymatic degradation of starch by limiting the functioning of amylase and glucosidase enzymes [7]. Moreover, tea polyphenols can influence the physicochemical characteristics of starch through interaction with it [8]. Phenolic compounds can interact with starch to form a V-type amylose inclusion complex or a non-inclusive complex, mainly formed through hydrogen bonds [9]. Research shows that tea polyphenols can inhibit starch retrogradation, and, during gelatinization, the phenolic polyhydroxy structure interacts with random helical starch chains with the aid of hydrogen bonding [10]. Furthermore, tea polyphenols also decrease the hydrolysis of starch [11]. Catechins constitute 70–80% of all tea polyphenols. Epigallocatechin gallate (EGCG) is the predominant compound among TPs, constituting about 59% of the overall content [12]. EGCG has multiple hydroxyl groups, which allow it to firmly attach to the active centers of α-amylase and α-glucosidase and to compete with starch [13]. Consequently, this leads to a reduction in starch digestibility and controls the postprandial blood glucose levels [14]. Research on the influence of tea polyphenols on starch digestibility has attracted increasing attention from experts and scholars. Although a wealth of literature focuses on how tea polyphenols inhibit starch hydrolysis and alter its physicochemical properties, few studies have analyzed their physicochemical properties or determined their digestibility after grinding with starch at different times.

Ball milling (BM) is an environmentally friendly processing technique that can alter the morphology and structure of starch granules through mechanical forces and friction. Studies have shown that starch chains can be broken under mechanical stress, resulting in a significant number of linear short chains [15]. Furthermore, ball milling has been demonstrated to facilitate the binding of starch with bioactive polysaccharides [7]. Thus, ball milling could be a potential method for the production of low-digestibility functional foods. Additionally, research on the preparation of complexes with anti-digestive properties using ball milling remains limited.

Therefore, we chose canna starch (CS), which has lower digestibility and is a potential raw material for the preparation of RS [16], and employed ball milling to modify the structure and properties of starch granules and promote its binding with tea polyphenols, thereby altering its physicochemical properties and enhancing its digestion resistance. Molecular dynamics (MD) simulation, as a computer simulation of molecular motion, has been applied in the study of the interactions of straight-chain starch with lipids, polyphenols, etc. [17]. While other substances of tea polyphenols may also interact with starch, we selected EGCG, the predominant component in tea polyphenols, as a model for MD simulations to investigate the interaction mechanisms between tea polyphenols and starch, as well as the effects of ball milling on these interactions. In this study, we systematically analyzed the effects of varying durations of ball milling treatment on the physicochemical properties of canna starch/tea polyphenol complexes, including their solubility, gelatinization characteristics, rheological properties, and thermodynamic properties. Additionally, we characterized the structural aspects of these complexes and explored the interactions between the starch and tea polyphenols with MD simulations. Exploring the interaction mechanisms between tea polyphenols and starch after ball milling provides a theoretical basis for the control of the content of resistant starch and the development of functional types of starch.

## 2. Materials and Methods

### 2.1. Materials and Instruments

Canna starch (with an amylose/amylopectin ratio of 0.72 ± 0.03 and granule size of 1217.9 ± 220.1 nm) was purchased from Guizhou Meirenyu Agricultural Development Co., Ltd., Qianxinan Buyi and Miao Autonomous Prefecture, Guizhou Province, China. Tea polyphenols (purity: 99.5%) were purchased from Shandong Yousuo Chemical Co., Ltd., Lanshan District, Linyi City, Shandong Province, China. All other chemicals used were of analytical grade, and deionized (DI) water was used throughout the experiment.

### 2.2. Preparation of Samples

Canna starch and tea polyphenols were weighed out in a 10:1 (*w*/*w*) ratio and evenly mixed to obtain a dry canna starch/tea polyphenol mixture. The mixture and steel balls were then placed into a ball mill (Grinder Corporation, Changping District, Beijing, China) at a weight ratio of 1:6 and ground for 1 h, 1.5 h, 2 h, 2.5 h, or 3 h at a grinding speed of 350 r/min. The samples were then sieved through a 100-mesh screen and set aside.

### 2.3. Determination of Solubility and Swelling Power

The determination was performed in accordance with the approach of Xie et al. [18], with minor modifications. A 0.5 g sample (m) awaiting testing was placed in a centrifuge tube, mixed with 30 mL of pure water, and heated in a water bath at 90 °C for 30 min with continuous stirring. The fully gelatinized sample was then centrifuged at 5000 rpm in a centrifuge for 30 min (Centrifuge, Eppendorf Corporation, Hamburg, Germany); the weight of the precipitate after centrifugation was the mass of swollen starch (m_1_). The supernatant obtained from centrifugation was transferred to a constant-weight aluminum box and dried to a constant weight at 105 °C. The mass of water-soluble starch was m_2_.

Solubility S (%) = (m_2_/m) × 100% (1)

Swelling power SP(g/g) = m_1_/(m − m_2_) (2)

(m: the weight of the sample/g; m_1_: the weight of the swollen starch/g; m_2_: the weight of the water-soluble starch/g).

### 2.4. Particle Size Analysis and Complex Particle Size Determination

Referring to the approach of Sun et al. [19], with minor modifications, the sample to be tested was crushed in a mortar and passed through a 100-mesh sieve. A 0.01 g amount of the sample was dispersed in 10 mL of pure water, shaken evenly, and then placed in a particle size analyzer (Zetasizer Nano ZS, Malvern Instruments Ltd., Enigma Business Park, Grovewood Road, Malvern, Worcestershire, UK) for analysis and determination.

### 2.5. Thermogravimetric Analysis (TGA)

According to the method of Kathyayani et al. [20], with some modifications, we dried the sample awaiting testing in a vacuum drying oven for 24 h for later use. Before the test, we turned on the nitrogen for about 30 min and waited for the flow rate to stabilize. We weighed 10–12 mg of the sample into a platinum crucible. The settings were as follows: starting temperature 25 °C, ending temperature 600 °C, heating rate 10 °C/min, nitrogen flow rate 25 mL/min (TGA55 Thermogravimetric Analyzer, TA Company, New Castle, DE, USA).

### 2.6. Determination of Starch Gelatinization Properties

The pasting properties were determined with reference to the method of Pang et al. [21]. We weighed 3.00 g starch, added 25 mL deionized water, mixed it in an aluminum box, and stirred thoroughly; we used a rapid viscosity analyzer (RVA Fast Viscosity Analyzer, Perten Company, Warriewood, New South Wales, Australia) to determine the gelatinization properties. The following parameters were set: held at 50 °C for 2 min, heated to 95 °C at a rate of 12 °C/min, held for 2.5 min, reduced to 50 °C at the same rate, held for 2 min. The stirring rate was 960 r/min in the first 10 s and 160 r/min in the latter.

### 2.7. Rheological Properties

#### 2.7.1. Measurement of Static Rheological Properties

The rheological properties were determined with the slight modification of the method of Xie et al. [22]. We chose a plate with a diameter of 25 mm and set the parameters as follows: 25 °C, a gap of 1000 μm, and a shear rate of 0~300 s^−1^, using the freshly gelatinized sample in RVA for measurement. We used the power law (power law model) to regress the data together.

(Power law τ = Kγ^N^: τ is the shear stress/Pa; K is the consistency coefficient/(Pa·s^n^); γ is the shear rate/s^−1^; N is the fluid index; correlation coefficient r^2^).

#### 2.7.2. Determination of Dynamic Viscoelastic Properties

We chose a plate with a diameter of 25 mm and set the parameters as follows: gap 1000 μm, 25 °C, sweep strain value 1%, and oscillation frequency 0.1–10 Hz. We used the freshly gelatinized sample in RVA to determine the storage modulus (G′) and loss modulus (G″) changes with an angular frequency.

### 2.8. Fourier Transform Infrared (FT-IR) Spectra Collection

A Fourier transform infrared analyzer (Vector-22, PerkinElmer Spectrum100, PerkinElmer Inc., Waltham, MA, USA) was used for infrared spectroscopy analysis. The methodology referenced and was modified from Pourfarzad et al. [23]. We weighed 1 mg of the sample awaiting testing and 100 mg of potassium bromide into an agate mortar, evenly mixed the sample and potassium bromide, and then pressed the tablet. After measuring the background path, the sample was started. The signal was accumulated by scanning it 60 times, and the scanning range was 450–4000 cm^−1^.

### 2.9. X-Ray Diffraction (XRD)

Wide-angle X-ray diffraction patterns of the samples were obtained using an X-ray diffractometer (X’Pert3 Powder, PANalytical B.V., Almelo, The Netherlands). The patterns were recorded at 2θ values ranging from 3° to 40°, with a step size of 0.02° and a scanning rate of 4° per minute, all at room temperature.

### 2.10. Scanning Electron Microscopy (SEM)

We dispersed the dried sample on black conductive glue on a metal copper plate, blew off the excess sample particles, coated it with gold, and started the test [24]. We observed and took pictures at 500 and 1500 magnification under an acceleration voltage of 15 kV (S-3000N scanning electron microscope, Olympus, Tokyo, Japan).

### 2.11. MD Simulation

MD simulations were used to predict how the starch would bind to the tea polyphenols and to explain their interactions. The long-chain amylose (LCA) model, modeled by the Carbohydrate Builder tool (Woods Group. 2005–2023 GLYCAM Web. Complex Carbohydrate Research Center, University of Georgia, Athens, GA, USA; http://glycam.org accessed on 29 August 2023), was composed of 30 glucose residues, while short-chain amylose (SCA) was composed of 15 glucose residues per lefthanded helix that consisted of 6 glucose residues. EGCG was constructed using the Avogadro software (version 1.2.0) [25] and further optimized with the GAFF force field. The MD simulation was performed using GROMACS (version 2023.02) and the results were visualized using PyMOL (The PyMOL Molecular Graphics System, version 2.6.0a0 open-source, Schrödinger, LLC, New York, NY, USA). Firstly, structure and topology files for amylose chains were created using the AmberTools23 program with the LEaP and parameters from the GLYCAM_06j-1 force field [26]. Meanwhile, the GAFF force field was applied to generate topology and coordinate files for EGCG. Secondly, the EGCG was incorporated into a cubic box comprising one LCA and two SCAs, respectively, to simulate the process of ball milling for amylose disruption. The resulting systems were named LCA@EGCG and SCA1-SCA2@EGCG, and relevant controls, including free LCA and free SCA1-SCA2, were included in the study. In each system, the box was filled with TIP3P water, and the total charges were neutralized. Thirdly, each system was energy-minimized with a conjugate gradient method of 5000 steps and pre-equilibrated using the leapfrog algorithm under NVP and NPT conditions of 1 ns, respectively. Finally, all systems were simulated with a time step of 2 fs for 100 ns.

### 2.12. In Vitro Digestibility Determination

The evaluation of the experimental in vitro digestibility mainly referred to Englyst’s method [27], with slight modifications. The specific operation steps were as follows. A 200 mg sample and 15 mL pH 6.8 phosphate buffer solution were placed in an Erlenmeyer flask, equilibrated on a constant-temperature shaker at 37 °C and 120 r/min for 5 min, and then poured into a dialysis bag, while 10 mL α-amylase (280 U/mL) and 1 mL glucoamylase (2500 U/mL) were hydrolyzed for 0, 20, and 120 min. In addition, 1 mL was sampled to determine the glucose content based on the DNS method. The experiment was repeated 3 times and the average value was calculated. The content of fast digestion starch (RDS), slow digestion starch (SDS), and resistant starch (RS) was calculated according to the following formulas:

RDS (%) = (G_20_ − FG) × 0.9/TG (3)

SDS (%) = (G_120_ − G_20_) × 0.9/TG (4)

RS (%) = (1 − RDS − SDS) × 100(5)

(In the formulas, G_20_ is the glucose released after 20 min of enzymolysis, mg; G_120_ is the glucose released after 120 min of enzymatic hydrolysis, mg; FG is free glucose, mg; TG is the weight of total starch, mg; 0.9 is the coefficient of the conversion of glucose into starch).

### 2.13. Statistical Analysis

A minimum of three replicates were performed for all experiments. The results were expressed as the mean ± standard deviation (SD). Means were compared using one-way analysis of variance (ANOVA) and Duncan’s test (*p* < 0.05) as a post-hoc test using the SPSS software version 26.0 (SPSS Inc., Evanston, IL, USA).

## 3. Results and Discussion

### 3.1. Solubility and Swelling Power

**Figure 1:**
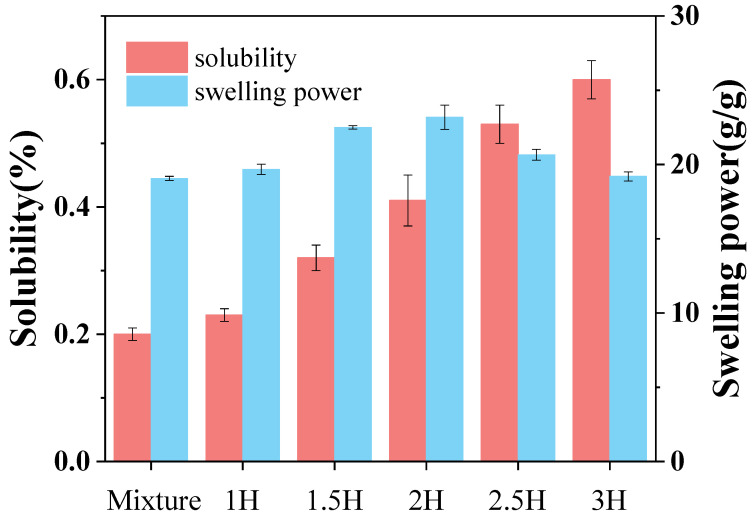
The solubility and swelling power of the TP–CS mixture without BM treatment (Mixture) and TP–CS complexes after BM treatment (1H, 1.5H, 2H, 2.5H, 3H).

As can be seen from Figure 1, there was no significant difference in the solubility of the complex milled for 1 h compared to the mixture. When the milling time was further extended, the solubility of the complex significantly increased (*p* < 0.05). After BM treatment for 3 h, its solubility increased by 199.4% compared to the untreated mixture. The main reason for this phenomenon under this condition may be that the mechanical force generated by the ball mill can destroy the crystalline structure of starch. At this time, the microcrystalline bundles of starch began to loosen, the polar groups were exposed, and the strong, water-soluble TPs formed a complex with the starch; thus, the solubility of the ball-milled modified TP–CS complex increased significantly [28]. Differing from the solubility, the swelling force of the TP–CS complex increased with the BM treatment time from 1 to 2 h. Beyond 2 h, however, the swelling force of the complex decreased. This is likely because, during the 1–2 h treatment period, the granular structure of the complex remained intact, and BM reduced the particle size of the complex, promoting water absorption and thereby enhancing the swelling force. When the BM treatment exceeded 2 h, the starch granule structure was disrupted. As the BM time increased, the particle fragmentation intensified, and the crystalline structure of the complex was progressively damaged, leading to a decrease in the complex’s swelling force.

### 3.2. Particle Size Determination of Complexes

**Figure 2:**
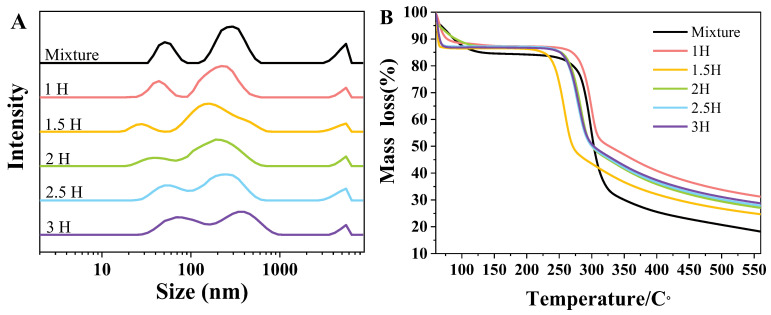
The particle size distribution (**A**) and thermogravimetric analysis (**B**) of the TP–CS mixture without BM treatment (Mixture) and TP–CS complexes after BM treatment (1H, 1.5H, 2H, 2.5H, 3H).

Figure 2A shows the particle sizes of the complexes with different BM times. After the starch granules were mechanically damaged, their particle size changed significantly. Theoretically, as the milling time lengthens, the large starch granules should gradually decrease and the small granules should increase. However, the experimentally measured particle size of the compound after ball milling decreased significantly within 0–1.5 h of ball milling, and it began to increase after 2 h, as ball milling damaged the canna starch particles and caused them to produce more agglomerates with TPs. This observation aligns with the phenomenon observed under scanning electron microscopy, suggesting that the mechanical effect of ball milling on starch represents a dynamic equilibrium process during which material refinement and complexation occur concurrently.

### 3.3. TGA

Endothermic volatilization during the thermal process caused changes in the weight of the TP–CS complexes [29]. After 250 °C, the weight loss of the compound mainly resulted from the thermal degradation of the TP–CS complex. Elevated temperatures can disrupt the chemical bonds within compounds, thereby compromising their structures. Aerobic conditions result in a small organic compound forming during decomposition, molecular evaporation, and sublimation [30]. The TP–CS complex exhibited two weight loss stages after 60–150 °C and 250–300 °C (Figure 2B). The initial weight loss (before 150 °C) was due to the evaporation of water, and the second stage was primarily due to the pyrolysis of starch, as starch constituted the majority (90.91%) of the complex, and the molecular weight of tea polyphenols is relatively small, resulting in an inconspicuous pyrolysis effect [31].

A comparison of the weight loss rates of the TP–CS complexes at 550 °C revealed that BM treatment could significantly improve the thermal stability of the complexes. The thermal stability of the complex with 1 h was the highest, while that at 1.5 h was the lowest. At 1 h, the CS structure remained intact, and extrusion promoted the partial combination of the starch and tea polyphenols, giving the complex higher thermal stability. When the BM time was increased to 1.5 h, the starch particles broke and exposed more hydroxyl groups, leading to greater weight loss during the heating process due to the evaporation of water resulting from the condensation of the hydroxyl groups. The 1.5–3 h group’s thermal stability increased; this may have been due to the further enhancement of the hydrogen bond interactions between the TPs and CS, which led to a reduction in the number of hydroxyl groups within the molecular structure. As a result, fewer hydroxyl groups were involved in condensation reactions during the thermal process, which in turn promoted the thermal stability of the complex, consistent with the results of the particle size determination shown in Figure 2A. Therefore, after the co-milling treatment, the thermal stability of the ball-milled starch with TP improved, likely due to the interaction between the starch and TPs during ball milling.

### 3.4. Gelatinization Characteristics

The changes in the gelatinization characteristics are given in Table 1. Compared to the original mixture, the peak viscosity, valley viscosity, and final viscosity of the TP–CS complex after BM treatment at different times were reduced, with the reductions positively correlating with the milling time. After ball milling for 3 h, these three parameters of the TP–CS complex decreased by 97.15%, 94.78%, and 93.01%, respectively. Furthermore, it is worth noting that, after BM treatment for 3 h, the complex disintegration value and retrogradation value decreased by 99.49% and 73.97%, respectively. The decrease in the pasting viscosity with the increase in the ball milling time was in line with the results presented by Shen et al. [32]. This suggests that mechanical forces such as impact and shear resulting from BM can destroy the original crystal structure of the complex by breaking the covalent bond and amorphizing it, thereby reducing its flow resistance. The reduced disintegration value indicates a decrease in the shear resistance of the TP–CS complex and an increase in the thermal paste. The significant decrease in the retrogradation value indicates that BM treatment degrades canna starch amylopectin into linear chains, and it also destroys the hydrogen bonds between amylose and amylopectin, increasing the content of RS in canna starch.

### 3.5. Static Rheological Analysis

**Figure 3:**
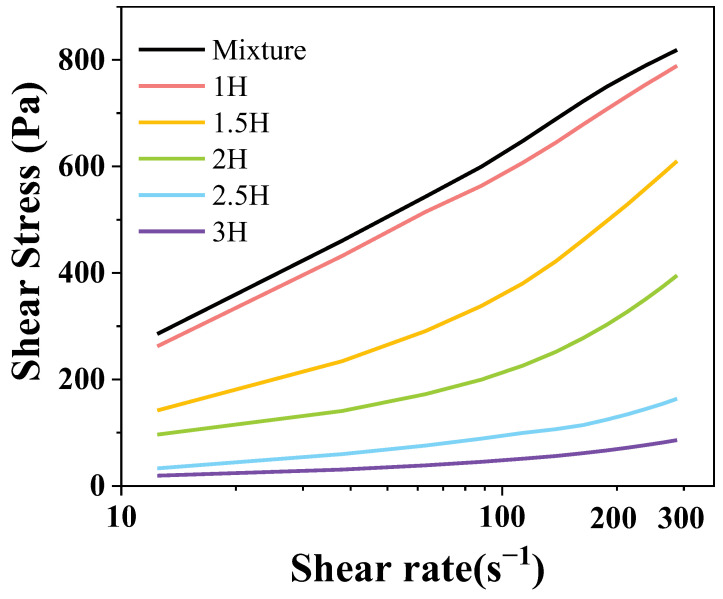
The flow characteristics of the TP–CS mixture without BM treatment (Mixture) and TP–CS complexes after BM treatment (1H, 1.5H, 2H, 2.5H, 3H).

After BM treatment, the paste rheological curve of the TP–CS complex changed significantly, as shown in Figure 3. With the increasing shear rate, the shear stress rose rapidly in its early stage and flattened in its later stage, indicating a pseudo-plastic fluid characteristic. When the shear rate was 300 s^−1^, compared with the original complex, after 1 h, 1.5 h, 2 h, 2.5 h, and 3 h of BM treatment, the complex shear rate decreased by 3.64%, 25.52%, 51.71%, 80.01%, and 89.48%, respectively. The results show that the shear stress at 1.5 h–2.5 h BM changed dramatically, but, after 2.5 h, the effect weakened, and the ball milling time did not linearly correlate with the TP–CS complex. Similar trends of change were also observed in starch that had been treated with high hydrostatic pressure under different pressures and numbers of cycles. BM treatment disrupts the hydrogen bonds of amylose, reduces the internal entanglement points, loosens the structure, and reduces the viscosity resistance, which in turn reduces the degree of shear thinning [33]. Furthermore, with increased processing time, the stronger mechanical force led to the greater micronization of the TP–CS complex, decreased its flow resistance, and lowered the shear stress.

The power law τ = Kγ^N^ was used to perform regression fitting on the data. The parameters are shown in Table 2. Among them, r^2^ was between 0.989 and 0.999, indicating that the power law could fit the TP–CS complex’s logistics curve appropriately. Furthermore, N fluctuated at around 0.432, which shows that the TP–CS complexes before and after treatment were pseudo-plastic fluids [34]. Meanwhile, K decreased as the homogeneous pressure increased, which was the combined effect of collision and shear resulting from ball milling. As the ball milling time increased, the probability that the particles would be subjected to mechanical forces increased. After ball milling for 2.5 h, the decrease in the viscosity coefficient was no longer significantly correlated with the change in the ball milling time.

### 3.6. State of Viscoelastic Properties Analysis

**Figure 4:**
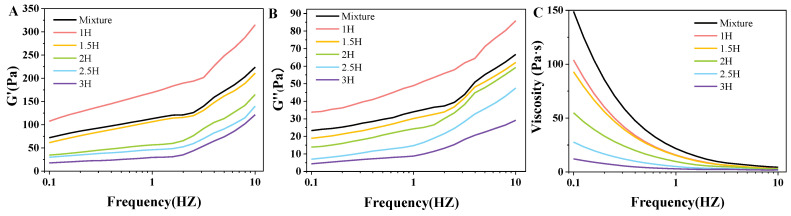
The storage modulus (**A**), loss modulus (**B**), and viscosity (**C**) of the TP–CS mixture without BM treatment (Mixture) and TP–CS complexes after BM treatment (1H, 1.5H, 2H, 2.5H, 3H).

Measuring the viscoelastic index of the gelatinized TP–CS complex in dynamic low-frequency scanning via the storage modulus (G′) and loss modulus (G″) and comparing their values served to provide frequency-related viscoelastic properties. As illustrated in Figure 4, G′ was greater than G″ under all treatment conditions, indicating that the TP–CS complex system was mainly elastic. There was no crossover between G′ and G″, which indicates that the TP–CS complex paste before and after treatment was in a weak gel state, consistent with the characteristics of a viscoelastic fluid.

**Figure 5:**
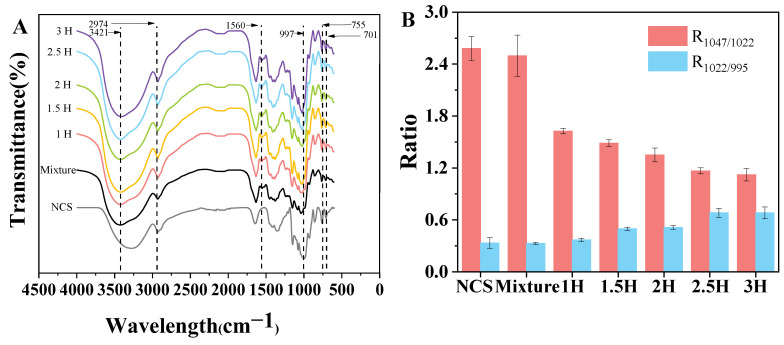
The FT-IR spectrum (**A**) and the short-range ordered degree (R1045/1022, R1022/995) (**B**) of native canna starch (NCS), the TP–CS mixture without BM treatment (Mixture), and the TP–CS complexes after BM treatment (1H, 1.5H, 2H, 2.5H, 3H).

After the ball milling treatment, as the ball milling time increased, G′ and G″ both decreased, indicating that ball milling reduced the elastic and viscous components in the TP–CS complex [35]. After the treatment, the elasticity of the TP–CS complex was weakened during deformation, the ability to restore the original state was reduced, and G′ was reduced. The increase in the ball milling time also reduced the viscosity of the starch paste (Figure 4C), and the flowability was enhanced. This may have been due to the ball milling treatment breaking the hydrogen bonds of the linear molecules in the canna starch, which is verified by the narrowing of the O/H characteristic band of the FT-IR spectrum in Figure 5A. As the ball milling time increased, the G′ and G″ of the CS–TP complex decreased, the crystal and helical structures of the starch were continuously destroyed, the intermolecular and intramolecular interactions of the starch were weakened, and the gel network structure was loosened. This structure was weak and its viscoelasticity was reduced.

### 3.7. FT-IR Spectroscopy

**Figure 6:**
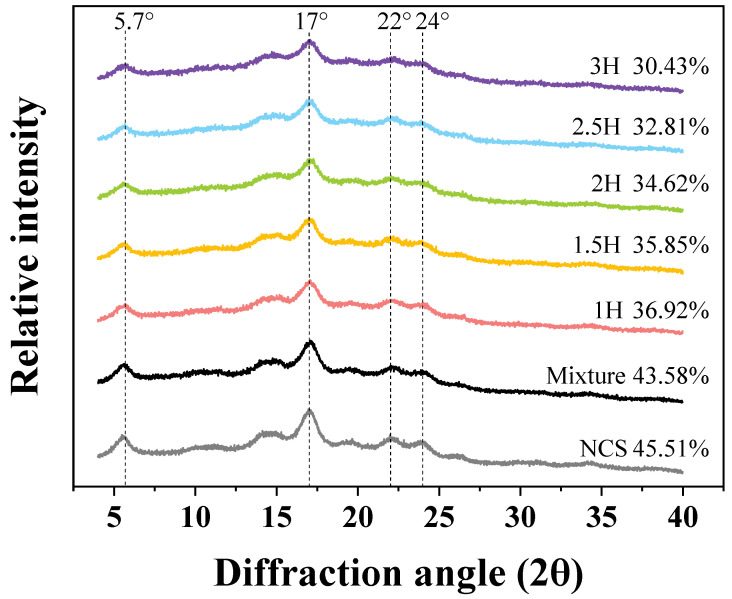
X-ray diffraction spectra of native canna starch (NCS), the TP–CS mixture without BM treatment (Mixture), and the TP–CS complexes after BM treatment (1H, 1.5H, 2H, 2.5H, 3H).

The FT-IR spectra of the TP–CS complexes treated with different milling times are shown in Figure 5A. The characteristic band at 3421 cm^−1^ is attributed to the stretching vibration of -OH and related to the intra- and intermolecular hydrogen bonds; as the milling time increased, the broad absorption band was narrowed, indicating that the content of hydroxyl groups decreased [36]. Near the wavenumber of 2974 cm^−1^ is the absorption shock band produced by -CH2. In the samples with added TPs, the absorption band at 1560 cm^−1^ can be attributed to the C=O stretching of tea polyphenols [37], while this band is not present in native canna starch. In addition, 997 cm^−1^ is the bending shock absorption band of the hydroxyl group, while 701 cm^−1^ and 755 cm^−1^ are the characteristic bands of monosubstituted benzene (C-H out-of-plane bending) [38]. The changes in the above characteristic bands indicate that, during ball milling, the TPs interacted with the canna starch; ball milling destroyed the crystal structure of the starch granules (as shown in Figure 6), which increased the exposure of the hydroxyl groups in the starch chain. TPs may be sandwiched between starch chains through hydrogen bonds; the conjecture about this binding mode was verified in the prediction of the conformations from the molecular dynamics simulations.

The characteristic bands in the 800~1200 cm^−1^ segment were deconvoluted, and absorbance ratios of 1047/1022 cm^−1^ (R1047/1022) and 1022/995 cm^−1^ (R1022/995) were observed, as shown in Figure 5B. R1047/1022 and R1022/995 are associated with the short-range ordered structure of starch samples. The former is positively correlated with its degree of ordering, while the latter is negatively correlated with its degree of ordering. The R1047/1022 of the TP–CS complexes treated with 1, 1.5, 2, 2.5, and 3 h of ball milling were 1.62, 1.48, 1.34, 1.16, and 1.12, respectively, which were lower than that of the untreated BM mixture (2.49). Moreover, the values of R1047/1022 decreased gradually with the increase in the ball milling time. In addition, R1022/995 increased with a prolonged ball milling time, which could be attributed to the fact that the impact, shear, and friction generated by ball milling disrupted the double helix structure of the starch molecules, thus reducing their short-range ordered structure.

### 3.8. XRD

In the XRD patterns, as depicted in Figure 6, native canna starch exhibited a characteristic B-type crystalline structure, with a distinct peak at 2θ ≈ 5° and a strong peak at 17°, which is in agreement with previous reports [39]. Compared to the native canna starch, the crystalline peak values of starch mixed with tea polyphenols remain essentially unchanged. However, the crystalline peaks of the starch subjected to ball milling treatment are significantly reduced, with the double peaks at 2θ = 22° and 24° nearly vanishing, and the intensity of the peaks associated with crystallinity decreases with prolonged ball milling. The degree of crystallinity index was calculated for each sample analyzed and is presented in Figure 6. The crystallinity of the starch–tea polyphenol mixture (43.58%) is slightly lower than that of native canna starch (NCS, 45.51%), and, as the ball milling time increases, the crystallinity of the starch decreases to a minimum of 30.43%. This indicates that the crystalline regions of the starch are more severely disrupted, and the interaction between the tea polyphenols and starch inhibits the aggregation of the starch molecules. This result is consistent with the trend of changes in the gelatinization viscosity with the ball milling time.

### 3.9. SEM

**Figure 7:**
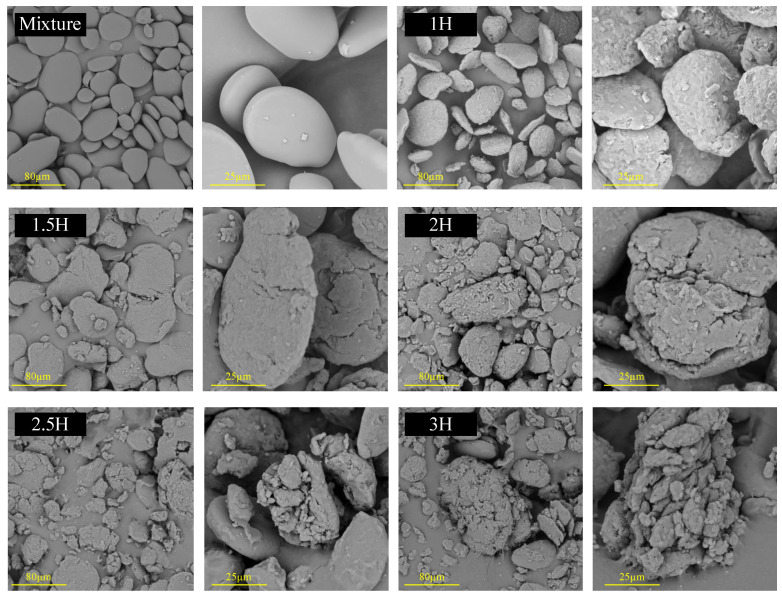
The SEM images (×500 on the left and ×1500 on the right) of the TP–CS mixture without BM treatment (Mixture) and TP–CS complexes after BM treatment (1H, 1.5H, 2H, 2.5H, 3H).

Figure 7 shows the SEM images of the TP–CS complex after different milling times. Canna starch exhibits a smooth, oval shape, while tea polyphenols appear as irregularly shaped small molecules. The general morphological characteristics of the TP–CS complex closely resemble those of CS. After BM for 1 h, the surface of CS becomes rough but maintains its original structure. After 1.5 h, with increased BM time, the particle shapes gradually become irregular and fragmented, with the small TP molecules becoming embedded with the CS particles; after 2 h of BM, small mixed particles start to aggregate around the CS particles; and as the BM treatment reaches 3 h, the particles develop more numerous and deeper cracks. At this point, the small molecules of tea polyphenols form complexes with the canna starch fragments. The figure shows that the ball-milled modified starch features a rough surface and exhibits significant deformation, including the formation of cracks and surface depressions. This deformation significantly increases the specific surface area of the starch, thereby greatly enhancing the adsorption capacity of the starch modified by ball milling.

### 3.10. Structural Stability Analysis

**Figure 8:**
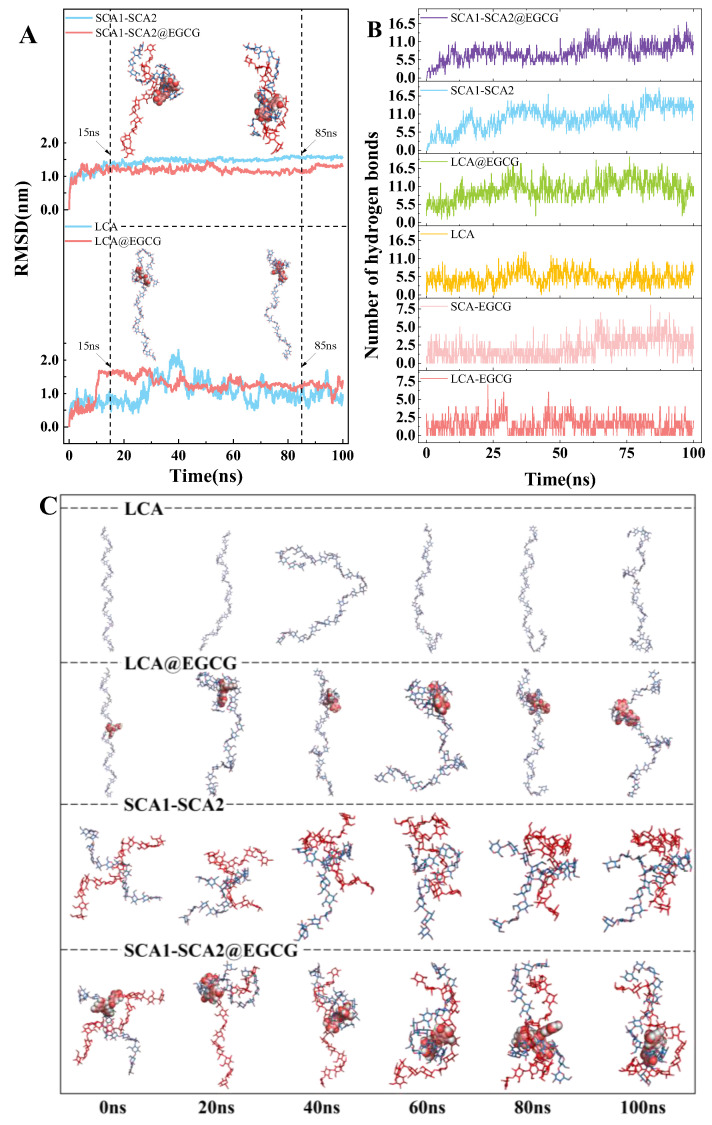
The RMSD (**A**), the number of hydrogen bonds (**B**), and the conformational changes and snapshots (**C**) of the free LCA, free SCA1-SCA2, LCA@EGCG, and SCA1-SCA2@EGCG complexes (representative MD trajectories for the LCA@EGCG and SCA1-SCA2@EGCG systems at 15 and 85 ns are shown in the graph (**A**), the red and blue stick models represent amylose, while the white and red ball models represent EGCG in the graph (**C**)).

A system containing one LCA or two SCAs was employed to simulate the interactions between starch and tea polyphenols before and after the ball milling process. The root mean square deviation (RMSD) values were calculated for the free LCA, free SCA1-SCA2, LCA@EGCG, and SCA1-SCA2@EGCG complexes to assess their structural stability during the simulation. As depicted in Figure 8A, the RMSD values of the free LCA, free SCA1-SCA2, LCA@EGCG, and SCA1-SCA2@EGCG complexes rose to approximately 0.90 nm, 1.62 nm, 1.36 nm, and 1.18 nm, respectively, within the first 15 ns. Thereafter, the RMSD value of LCA fluctuated significantly due to its long molecular chain flexibility. The RMSD value of the SCA1-SCA2@EGCG complex fluctuated within a range of 2 Å, averaging 1.21 ± 0.08 nm, which was lower than that of the LCA@EGCG complex (1.30 ± 0.17 nm), indicating that BM treatment caused amylose breakage, thereby facilitating starch binding with EGCG. The RMSD value of the SCA1-SCA2@EGCG complex was also lower than that of the free SCA1-SCA2 system (1.49 ± 0.07 nm), suggesting that EGCG could enhance the stability in this system. Therefore, the results indicate that the MD simulation trajectory can be used for subsequent analysis after equilibration.

### 3.11. Hydrogen Bond Dynamics

The number of hydrogen bonds formed between long-/short-chain amylose and EGCG is shown in Figure 8B. Once stability is achieved, the LCA-EGCG complex exhibits interchain hydrogen bonds fluctuating between 0 and 6, whereas the SCA-EGCG complex shows a range of 0–8. Notably, the significant increase in the frame number with hydrogen bond numbers > 6 indicates that the BM-induced breakage of amylose results in more short-chain amylose forming additional hydrogen bonds with EGCG, which aligns with the findings of the FTIR analysis. Furthermore, we also calculated the number of intramolecular hydrogen bonds within amylose in various systems, including free LCA, free SCA1-SCA2, LCA@EGCG, and SCA1-SCA2@EGCG. In the free LCA system, intramolecular hydrogen bonds ranged from 0 to 13. However, in the presence of EGCG, these fluctuations extended from 2 to 20, attributed to EGCG’s ability to reduce the pitch length of LCA and promote intramolecular hydrogen bond formation, as observed in Figure 8C, where the EGCG molecule is encapsulated within the spiral structure of amylose. Without the involvement of EGCG, the amylose chains in the free SCA1-SCA2 system intertwine with each other to form a double helix structure, with the number of intramolecular hydrogen bonds varying between 3 and 19. The SCA1-SCA2@EGCG system shows a decrease in the number of hydrogen bonds within SCA molecules, fluctuating within the range of 3–17.

### 3.12. Energy Contribution Analysis

**Figure 9:**
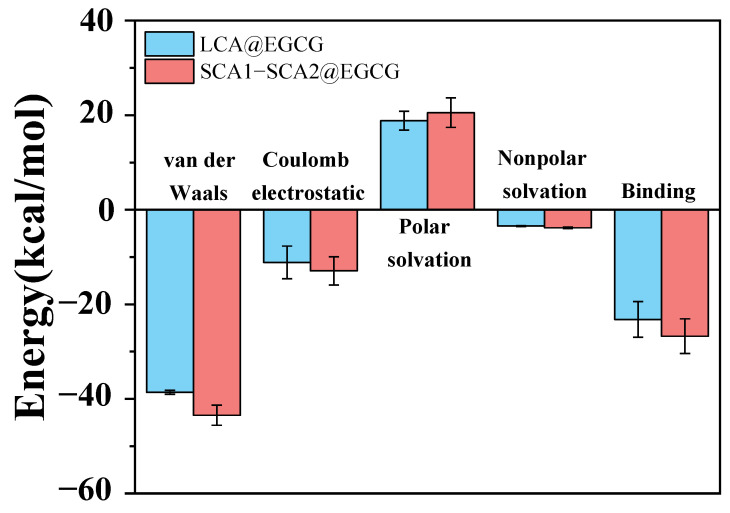
The energy contributions of the LCA@EGCG and SCA1-SCA2@EGCG complexes.

Figure 9 illustrates the breakdown of the energy contributions. The binding energies of the LCA@EGCG and SCA1-SCA2@EGCG complexes are negative (−23.20 ± 3.77 and −26.73 ± 3.67 kcal/mol, respectively), which is beneficial for the formation of the complex. The SCA1-SCA2@EGCG complex has a lower binding energy, indicating a more robust interaction between EGCG and SCA1-SCA2. Moreover, the van der Waals energy significantly contributes to the binding energy, highlighting the pivotal role of van der Waals forces in maintaining the stability of the complex. The Coulomb electrostatic energy also plays a significant role in the binding energy. Based on the MD simulation results, the hydrogen bond is primarily electrostatic in nature. Furthermore, the decrease in the absorption peak area corresponding to the stretching vibration of hydroxyl groups in the FTIR spectrum with increasing ball milling time suggests a strong hydrogen bond interaction between the components.

### 3.13. In Vitro Digestibility of TP–CS Complex

**Figure 10:**
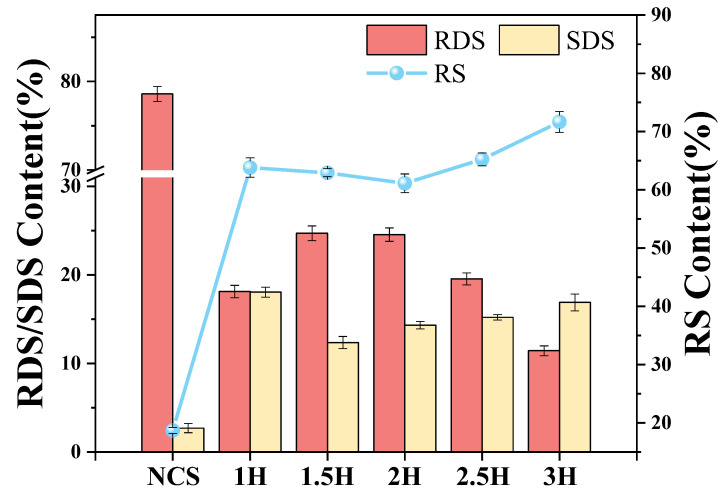
The digestion characteristics of the TP–CS mixture without BM treatment (Mixture) and TP–CS complexes after BM treatment (1H, 1.5H, 2H, 2.5H, 3H) (RDS, rapidly digestible starch; SDS, slowly digestible starch; RS, resistant starch).

The variation in the in vitro digestibility of the TP–CS complexes with the ball milling time is shown in Figure 10. The incorporation of TPs significantly enhanced the RS content in the starch compared to natural canna starch (NCS) by 226.84% to 283.21%. With an increased ball milling treatment time, the RDS content of the TP–CS compound initially increased and then decreased, while the RS content first decreased and then increased. This is attributed to the micronization effect of ball milling and the complexation of TPs. Ball milling disrupts the original crystalline structure of the complex, transforming it into an amorphous state, thereby increasing the interaction sites of enzymes and starch [40], making the post-ball milling starch easier to digest than native starch.

Simultaneously, the ball milling treatment disrupted the spatial arrangement of the starch and destroyed the crystallization area of the starch granules, which can contribute to the formation of complexes between starch and TPs. Furthermore, the formation of starch–tea polyphenol complexes mediated by hydrogen bonds can promote the aggregation of starch, leading to reduced substrate exposure to enzymes, and starch exhibits the characteristics of slow digestion. Zhang et al. [41] showed that the digestion of starch was mainly influenced by digestive enzyme activity. TPs can inhibit the digestibility of starch since hydrogen bonds form between its hydroxyl groups and digestive enzymes; under the combined action of hydrophobic interactions and hydrogen bonds, TPs form complexes with digestive enzymes, thereby reducing the catalytic activity of the enzymes.

## 4. Conclusions

An extended ball milling time alters the original particle structure and morphology of TP–CS complexes, intensifying the degree of fragmentation. This results in increased surface energy, significantly enhancing the interactions between canna starch and tea polyphenols. Ball milling conditions cause the starch microcrystal bundles to loosen and amylopectin to degrade into amylose, increasing the content of slow-digested starch. MD simulations demonstrate a molecular mechanism for the interaction between tea polyphenols and starch. The simulations indicate that ball milling leads to starch fragmentation, consequently strengthening the interaction between EGCG and amylose. The primary site of interaction with amylose is the B ring of EGCG. Under the combined effect of van der Waals forces and hydrogen bonding, tea polyphenols form complexes with starch, inhibiting interactions between starch. This modifies the physicochemical properties of starch and enhances the anti-digestibility of starch. In summary, canna starch and tea polyphenols after ball milling exhibit excellent physicochemical properties and display good anti-digestive properties.

## Figures and Tables

**Table 1:** Effects of ball milling treatment on the gelatinization properties of TP–CS complexes ^a^.

Sample	PV (cP)	TV (cP)	BD (cP)	FV (cP)	SB (cP)
Mixture	5738.3 ± 17.2 ^a^	3012.5 ± 26.0 ^a^	2661.7 ± 24.9 ^a^	3122.3 ± 25.2 ^b^	110.7 ± 1.5 ^c^
1H	3608.2 ± 16.2 ^b^	2473.3 ± 8.6 ^b^	1129.3 ± 8.5 ^b^	3218.0 ± 17.0 ^a^	748.7 ± 21.5 ^a^
1.5H	1661.3 ± 16.9 ^c^	1234.3 ± 12.9 ^c^	427.0 ± 12.5 ^c^	1751.0 ± 14.7 ^c^	526.7 ± 22.3 ^b^
2H	892.7 ± 13.7 ^d^	780.3 ± 10.9 ^d^	115.3 ± 9.0 ^d^	1105.7 ± 11.1 ^d^	335.3 ± 19.1 ^c^
2.5H	289.3 ± 9.1 ^e^	270.0 ± 7.0 ^e^	28.3 ± 1.5 ^e^	402.3 ± 8.0 ^e^	136.0 ± 4.6 ^d^
3H	163.3 ± 8.0 ^f^	157.3 ± 6.8 ^f^	13.7 ± 2.4 ^e^	218.3 ± 5.6 ^f^	88.7 ± 6.1 ^e^

^a^ Values are expressed as means ± standard deviation (*n* = 3). Values in the same column denoted with different superscripts are significantly different (*p* ≤ 0.05). (PV, peak viscosity; TV, valley viscosity; BD, disintegration value; FV, final viscosity; SB, regeneration value).

**Table 2:** Fitting parameters of ball milling treatment to TP–CS complex’s flow characteristics ^a^.

Sample	K (Pa·s^n^)	N	r^2^
Mixture	112.93 ± 4.51 ^b^	0.35 ± 0.03 ^d^	0.967
1H	133.49 ± 3.28 ^a^	0.31 ± 0.01 ^c^	0.995
1.5H	47.97 ± 4.58 ^c^	0.45 ± 0.02 ^b^	0.997
2H	28.67 ± 0.90 ^d^	0.45 ± 0.04 ^b^	0.983
2.5H	10.64 ± 1.13 ^e^	0.49 ± 0.01 ^a^	0.996
3H	6.90 ± 0.99 ^e^	0.47 ± 0.02 ^ab^	0.988

^a^ Values are expressed as means ± standard deviation (*n* = 3). Values in the same column denoted with different superscripts are significantly different (*p* ≤ 0.05).

## Data Availability

The raw data supporting the conclusions of this article will be made available by the authors on request.

## References

[1] Bai Y., Zhou X., Zhan C., Ma L., Yuan Y., Wu C., Chen M., Chen G., Ni Q., Wu F. (2017). 3D Hierarchical Nano-Flake/Micro-Flower Iron Fluoride with Hydration Water Induced Tunnels for Secondary Lithium Battery Cathodes. Nano Energy.

[2] Wang L., Ma R., Tian Y. (2024). Quercetin Slow-Release System Delays Starch Digestion via Inhibiting Transporters and Enzymes. Food Chem..

[3] Bodjrenou D.M., Li X., Lu X., Lei S., Zheng B., Zeng H. (2023). Resistant Starch from Sweet Potatoes: Recent Advancements and Applications in the Food Sector. Int. J. Biol. Macromol..

[4] Althawab S.A., Amoako D.B., Annor G.A., Awika J.M. (2023). Stability of Starch-Proanthocyanidin Complexes to in-Vitro Amylase Digestion after Hydrothermal Processing. Food Chem..

[5] García-Pérez P., Giuberti G., Sestili F., Lafiandra D., Botticella E., Lucini L. (2023). The Functional Implications of High-Amylose Wholegrain Wheat Flours: An in Vitro Digestion and Fermentation Approach Combined with Metabolomics. Food Chem..

[6] Zhu J., Tao K., Prakash S., Zhang C., Gilbert R.G., Liu Q. (2023). Using Starch Structure to Choose Rices with an Optimal Combination of Palatability and Digestibility. Food Hydrocoll..

[7] Sun L., Wang Y., Miao M. (2020). Inhibition of α-Amylase by Polyphenolic Compounds: Substrate Digestion, Binding Interactions and Nutritional Intervention. Trends Food Sci. Technol..

[8] Zhu F. (2015). Interactions between Starch and Phenolic Compound. Trends Food Sci. Technol..

[9] Zeng X., Zheng B., Xiao G., Chen L. (2022). Synergistic Effect of Extrusion and Polyphenol Molecular Interaction on the Short/Long-Term Retrogradation Properties of Chestnut Starch. Carbohydr. Polym..

[10] Li L., Gao J., Koh H.S.A., Zhou W. (2023). Bioaccessibility and Bioavailability of (-)-Epigallocatechin Gallate in the Bread Matrix with Glycemic Reduction. Foods.

[11] Xu C., Zhou S., Song H., Hu H., Yang Y., Zhang X., Ma S., Feng X., Pan Y., Gong S. (2023). Green Tea Polyphenols-Derived Hybrid Materials in Manufacturing, Environment, Food and Healthcare. Nano Today.

[12] Forester S.C., Gu Y., Lambert J.D. (2012). Inhibition of Starch Digestion by the Green Tea Polyphenol, (−)-Epigallocatechin-3-Gallate. Mol. Nutr. Food Res..

[13] Barber E., Houghton M.J. (2021). Flavonoids as Human Intestinal α-Glucosidase Inhibitors. Foods.

[14] Lv Y., Zhang L., Li M., He X., Hao L., Dai Y. (2019). Physicochemical Properties and Digestibility of Potato Starch Treated by Ball Milling with Tea Polyphenols. Int. J. Biol. Macromol..

[15] Bangar S.P., Singh A., Ashogbon A.O., Bobade H. (2023). Ball-Milling: A Sustainable and Green Approach for Starch Modification. Int. J. Biol. Macromol..

[16] Li H., Wang N., Zhang D., Wu J., Tan S., Li Y., Zhang N., Yang L., Wang X. (2024). Comparative Study on the Structure Characterization and Activity of RS5 Made from Canna Edulis Native Starch and High-Amylose Corn Starch. Int. J. Biol. Macromol..

[17] Chen N., Feng Z.J., Gao H.X. (2022). Effects of Phenols with Different Structure Characteristics on Properties of Potato Starch: Action Rule and Molecular Mechanism. J. Food Process. Preserv..

[18] Xie Q., Liu X., Liu H., Zhang Y., Xiao S., Ding W., Lyu Q., Fu Y., Wang X. (2023). Insight into the Effect of Garlic Peptides on the Physicochemical and Anti-Staling Properties of Wheat Starch. Int. J. Biol. Macromol..

[19] Sun C., Hu Y., Zhu Z., He Z., Mei L., Wang C., Xie Q., Chen X., Du X. (2024). Starch Nanoparticles with Predictable Size Prepared by Alternate Treatments of Ball Milling and Ultrasonication. Int. J. Biol. Macromol..

[20] Kathyayani D., Mahesh B., Channe Gowda D., Sionkowska A., Veeranna S. (2023). Investigation of Miscibiliy and Physicochemical Properties of Synthetic Polypeptide with Collagen Blends and Their Wound Healing Characteristics. Int. J. Biol. Macromol..

[21] Pang Z., Bourouis I., Sun M., Cao J., Liu P., Sun R., Chen C., Li H., Liu X. (2022). Physicochemical Properties and Microstructural Behaviors of Rice Starch/Soy Proteins Mixtures at Different Proportions. Int. J. Biol. Macromol..

[22] Xie F., Zhang H., Xia Y., Ai L. (2020). Effects of Tamarind Seed Polysaccharide on Gelatinization, Rheological, and Structural Properties of Corn Starch with Different Amylose/Amylopectin Ratios. Food Hydrocoll..

[23] Pourfarzad A., Yousefi A., Ako K. (2021). Steady/Dynamic Rheological Characterization and FTIR Study on Wheat Starch-Sage Seed Gum Blends. Food Hydrocoll..

[24] Zhu Q., Tang J., Yao S., Feng J., Mi B., Zhu W., Chen Q., Liu D., Xu E. (2023). Controllable Structure of Porous Starch Facilitates Bioactive Encapsulation by Mild Gelatinization. Food Hydrocoll..

[25] Hanwell M.D., Curtis D.E., Lonie D.C., Vandermeersch T., Zurek E., Hutchison G.R. (2012). Avogadro: An Advanced Semantic Chemical Editor, Visualization, and Analysis Platform. J. Cheminform..

[26] Kirschner K.N., Yongye A.B., Tschampel S.M., González-Outeiriño J., Daniels C.R., Foley B.L., Woods R.J. (2008). GLYCAM06: A Generalizable Biomolecular Force Field. Carbohydrates. J. Comput. Chem..

[27] Englyst H.N.N., Kingman S.M.M., Cummings J.H.H. (1992). Classification and Measurement of Nutritionally Important Starch Fractions. Eur. J. Clin. Nutr..

[28] González L.C., Loubes M.A., Tolaba M.P. (2018). Incidence of Milling Energy on Dry-Milling Attributes of Rice Starch Modified by Planetary Ball Milling. Food Hydrocoll..

[29] da Cruz E.P., Pires J.B., dos Santos F.N., Fonseca L.M., Radünz M., Dal Magro J., Gandra E.A., da Rosa Zavareze E., Dias A.R.G. (2023). Encapsulation of Lemongrass Essential Oil into Cassava Starch Fibers for Application as Antifungal Agents in Bread. Food Hydrocoll..

[30] Amaraweera S.M., Gunathilake C., Gunawardene O.H., Fernando N.M., Wanninayaka D.B., Manamperi A., Dassanayake R.S., Rajapaksha S.M., Gangoda M., Fernando C.A.N. (2021). Preparation and Characterization of Biodegradable Cassava Starch Thin Films for Potential Food Packaging Applications. Cellulose.

[31] Wang J., Yang H., Luo L., Ye H., Xu H., Sun Y., Gong L., Yang H. (2024). Persimmon Leaf Polyphenols as Potential Ingredients for Modulating Starch Digestibility: Effect of Starch-Polyphenol Interaction. Int. J. Biol. Macromol..

[32] Shen H., Yu J., Bai J., Ge X., Liang W., Ospankulova G., Muratkhan M., Zhang G., Li W. (2022). Electron Beam Irradiation Regulates the Structure and Functionality of Ball-Milled Corn Starch: The Related Mechanism. Carbohydr. Polym..

[33] Zhang X., Wang C., Liu Z., Xue Y., Zhao Q., Shen Q. (2023). Four Stages of Multi-Scale Structural Changes in Rice Starch during the Entire High Hydrostatic Pressure Treatment. Food Hydrocoll..

[34] Chen N., Wang Q., Wang M.-X., Li N., Briones A.V., Cassani L., Prieto M.A., Carandang M.B., Liu C., Gu C.M. (2022). Characterization of the Physicochemical, Thermal and Rheological Properties of Cashew Kernel Starch. Food Chem. X.

[35] Park J., Choi H.W., Park J.D., Choi H.D., Hong J.S. (2024). Impact of Annealing and Incorporation of Vegetable Oils on Physicochemical and Rheological Properties of Wheat Starch. Int. J. Biol. Macromol..

[36] Xie S., Chen H., Jiang X., Zhou B., Guo Z., Zeng H., Zhang Y. (2023). Structural and Physicochemical Properties of a Chinese Yam Starch–Tea Polyphenol Complex Prepared Using Autoclave-Assisted Pullulanase Treatment. Foods.

[37] Romero Hernández H.A., Gutiérrez T.J., Tovar J., Bello-Pérez L.A. (2023). Complexation of Octenyl Succinic Anhydride-Esterified Corn Starch/Polyphenol-Rich Roselle (*Hibiscus sabdariffa* L.) Extract: Structural and Digestibility Features. Food Hydrocoll..

[38] Wei Y., Cheng F., Zheng H. (2008). Synthesis and Flocculating Properties of Cationic Starch Derivatives. Carbohydr. Polym..

[39] Lan X., Xie S., Wu J., Xie F., Liu X., Wang Z. (2016). Thermal and Enzymatic Degradation Induced Ultrastructure Changes in Canna Starch: Further Insights into Short-Range and Long-Range Structural Orders. Food Hydrocoll..

[40] Liu L., An X., Zhang H., Lu Z., Nie S., Cao H., Xu Q., Liu H. (2020). Ball Milling Pretreatment Facilitating α-Amylase Hydrolysis for Production of Starch-Based Bio-Latex with High Performance. Carbohydr. Polym..

[41] Zhang H., Jiang Y., Pan J., Lv Y., Liu J., Zhang S., Zhu Y. (2018). Effect of Tea Products on the in Vitro Enzymatic Digestibility of Starch. Food Chem..

